# Does Vaccine Confidence Mediate the Relationship between Vaccine Literacy and Influenza Vaccination? Exploring Determinants of Vaccination among Staff Members of Nursing Homes in Tuscany, Italy, during the COVID-19 Pandemic

**DOI:** 10.3390/vaccines11081375

**Published:** 2023-08-17

**Authors:** Francesca Collini, Guglielmo Bonaccorsi, Marco Del Riccio, Mario Bruschi, Silvia Forni, Giacomo Galletti, Fabrizio Gemmi, Francesca Ierardi, Chiara Lorini

**Affiliations:** 1 Quality and Equity Unit, Regional Health Agency of Tuscany, 50141 Florence, Italy; 2 Department of Health Sciences, University of Florence, 50134 Florence, Italy

**Keywords:** vaccine literacy, vaccine confidence, influenza vaccination, human influenza, nursing homes, mediation model

## Abstract

Background: Low coverage of influenza vaccination in nursing home (NH) staff may be attributed to factors such as vaccine confidence (VC) and vaccine literacy (VL). Our study aimed to evaluate the role of VL and VC in predicting the intention to get the influenza vaccine in a sample of employees of NHs in Tuscany, Italy. Methods: Data from staff members in Tuscany were collected using an online questionnaire that examined influenza vaccination history, intentions, demographic information, health status, and VL. Statistical analyses explored the relationships between VC, VL, and vaccination intentions. Results: The study included 1794 respondents, (86.3%) and assistants/aides (58.1%), with a median age of 46 years. The intention to get vaccinated was significantly higher among those with health risk conditions, and there was a positive association between VC and VL, specifically its interactive/critical component. The mediation analysis showed that VC completely mediated the relationship between VL and the intention to get vaccinated, with significant effects observed in different subgroups. Conclusions: VC is a key factor that mediates the effect of VL on vaccine intention. These results suggest that interventions aimed at improving VL alone may not be sufficient to increase vaccine uptake unless VC is also addressed.

## 1. Introduction

The World Health Organization (WHO) estimates that there are 3 to 5 million severe cases of influenza and about 290,000 to 650,000 deaths (mostly from respiratory complications) annually worldwide. In high-income countries, most deaths associated with influenza occur among people aged 65 or older [1]. In particular, elderly people residing in nursing homes (NHs) represent a very susceptible group both in terms of infection risk and serious health consequences [2]. To prevent severe disease or death, vaccination is the mainstay for influenza [1,3]; high vaccine coverage would reduce influenza-related mortality, prevent co-infection, and help reduce the burden on the healthcare system during the co-circulation of influenza viruses and severe acute respiratory syndrome coronavirus 2 (SARS-CoV-2) [1,4,5]. Annual influenza vaccination is recommended for older individuals and other target groups, such as healthcare workers (HCWs) and those who live with at-risk people [3,6]. The influenza vaccination rates among NH residents tend to be slightly higher compared to that of the general population [2,7]; however, older people may be insufficiently protected by vaccination due to the immunosenescence which accompanies aging [8]. Thus, influenza vaccination among NH employees—HCWs and other staff members—is an important indirect protection strategy [9]. Nevertheless, immunization rates among them remain low with respect to the international recommended targets [10,11,12]. Reasons for such low influenza vaccination uptake among staff members of NHs may include a wide range of causes. Previous studies have described the motivations underlying the intention to get the influenza vaccine. In particular, the desire to protect others (colleagues, NH residents, at-risk relatives, or cohabitants) or oneself (due to individual risk conditions) is reported as the main factor determining the choice of whether to get the vaccine [12,13]. Among the more distal and general determinants, vaccine confidence (VC) has been deeply investigated [12,14,15]. VC was included in the first model for vaccine hesitancy (VH) developed by the Strategic Advisory Group of Experts on Immunization (SAGE) and is defined as the trust in the effectiveness and safety of vaccines and trust in the healthcare system that delivers them [16]. It is crucial for maintaining high coverage rates and is a predictor of self-reported influenza vaccination uptake among the staff of NHs [13]. Vaccine literacy (VL) is increasingly gaining ground in the international scientific literature as well. The construct of VL was born from that of health literacy (HL), with the latter considered as a determinant of vaccination uptake [17,18]. VL can be considered not only as people’s knowledge, motivation, and skills to find, understand, and evaluate immunization-related information in order to make adequate immunization decisions [19] but also in developing a system with decreased complexity to communicate and offer vaccines as sine qua non of a functioning health system [20,21], as also reported by recent research aimed at finding a new, comprehensive definition of VL [22].

To the best of our knowledge, the relative weight of VC and VL in predicting vaccination uptake—or a proxy of it, such as the intention to get vaccinated—as well as the relationship between VC and VL have not been deeply investigated yet; in fact, no studies have evaluated these three aspects (VC, VL, and vaccination uptake/intention to get vaccinated) at the same time. On the other hand, a survey conducted in a sample of NHs in 2018 reported that: (i) VC and HL were significantly and positively correlated; (ii) through univariate analysis, either VC or HL predicted influenza vaccination uptake; and (iii) through multivariate analysis, VC emerged as the strongest predictor of influenza vaccination uptake [12]. These results suggest the need to deepen the relationship between the possible determinants of vaccination uptake in such a target group.

For these reasons, we have conducted a new study aimed at evaluating the role of VL and VC in predicting the intention to take up influenza vaccination in a sample of employees of Tuscan NHs. In particular, a mediation effect of VC while analyzing the role of VL as a predictor was hypothesized. Moreover, since individual or familial risk conditions are frequently reported as motivations to get the vaccine, the secondary aim of the study was to assess whether the role of VL and VC in predicting the intention to take up influenza vaccination changes when considering those risk factors.

## 2. Materials and Methods

The study adopted a cross-sectional design and was conducted according to the principles of the Declaration of Helsinki. The survey was conducted in August–September 2020 in Tuscany, where the Regional Health Agency of Tuscany introduced the study via email to the chief officers of each Tuscan NH, totaling around 300. Among them, 98 NHs chose to partake voluntarily. Within each NH, all staff members were included, regardless of their employment agreements, job responsibilities, or qualifications, as described in a previously published paper [23].

### 2.1. Questionnaire

An online questionnaire was used to collect individual, self-reported data on influenza vaccination in two different seasons (2018/19 and 2019/20) from staff members. In addition, intentions to get vaccinated in the 2020/21 season were collected, with response options including “not at all”, “slightly”, “fairly high”, and “very high” intention to get vaccinated. Demographic, educational, health (including data on chronic cardiovascular, renal, respiratory, and autoimmune diseases, as well as self-perceived health status), and social information (such as cohabitation with individuals at risk, such as children, elderly individuals, or those with chronic conditions) were also collected. The questionnaire also contained 14 items from the HLVa-IT (Health Literacy Vaccinale in Italiano—Health Literacy for Vaccination in the Italian language) for measuring VL (see below). To assess VC, responses to eight Likert-scale items were collected (see below).

Recruitment was performed in two phases. First, the research group shared the aim of the study and the link to the online questionnaire with the director of each Tuscan NH; then, the directors who joined the study shared the link to the online questionnaire with all of the staff members. Participation was voluntary for both the directors and the staff members.

### 2.2. HLVa-IT

The HLVa-IT is a self-rated measure of VL in adults, based on the Ishikawa test [24]. It is composed of 14 Likert-type items aimed at assessing functional VL (items 1–5), interactive/communicative VL (items 6–10), and critical VL (items 11–14), according to Nutbeam’s definition of HL domains (Supplementary File S1) [25]. Each answer contains four possible choices, with an associated score (for the functional items: 4—never, 3—rarely, 2—sometimes, and 1—often; for the interactive and the critical items: 1—never, 2—rarely, 3—sometimes, and 4—often). In the validation study, interactive and critical items appear to belong to the same domain (HLVa-IT-interactive/communicative/critical subscale, hereinafter HLVa-IT-ICC), while the functional items constitute another specific domain (HLVa-IT-functional subscale, hereinafter HLVa-IT-F). It was developed to measure VL in the general adult population [26] and was then also validated for the staff of NHs [23].

The HLVa-IT contains two filter questions. In particular, either before submitting the functional items or before the interactive/communicative and critical items, a filter question is included, in order to select the subjects who have had previous experience with written documents on vaccines and vaccinations (“have you ever read vaccine materials, such as leaflets or posters in doctors’ or public health units’ offices, recommending vaccinations?”) and those who have ever thought or have been advised to vaccinate themselves (“have you ever thought or been advised to vaccinate yourself against one or more diseases?”).

The total score in the HLVa-IT was obtained by calculating the mean value of the answers to each item (range: 1 to 4); similarly, the scores for each subscale (HLVa-IT-F and HLVa-IT-ICC) were calculated as the mean values of the answers included in each subscale [26]. A higher value corresponded to a higher VL level.

### 2.3. Vaccine Confidence Index

A vaccine confidence index (VCI) was computed according to the literature and previous studies conducted in Tuscany [12,26,27,28,29]. In particular, the following eight Likert-type statements included in the staff questionnaire were used:

1. Influenza is a serious illness (A_1_)
2. The influenza vaccine is effective (A_2_)
3. Healthcare workers must get vaccinated (A_3_)
4. By getting vaccinated, I protect people close to me from influenza (A_4_)
5. It is better to contract influenza than to get the vaccination (B_1_)
6. Influenza vaccines have serious side effects (B_2_)
7. Vaccines can cause influenza (B_3_)
8. Opposed to vaccination (B_4_)

For each statement, the participants were asked to declare their agreement or disagreement as follows: “totally agree” (score: 1); “partially agree” (score: 2); “partially disagree” (score: 3); “totally disagree” (score: 4). For the first four statements (A_1_–A_4_), the higher the Likert score, the better the propensity towards vaccines while, for the second four (B_1_–B_4_), the higher the Likert score, the lower the propensity.

The vaccine confidence index was calculated as follows:

$$VCI=\frac{\sum_{i=1}^{n}A_{n}}{\sum_{i=1}^{n}B_{n}}$$

where A_n_ is the scores of the first four statements, while B_n_ is those of the second four. 

### 2.4. Statistical Analysis

The data were summarized as the mean and standard deviation (SD) or the median and interquartile range (IQR) for continuous variables, while frequency and percentage were used for ordinal and nominal variables. A new dichotomous variable on individual or familiar risk conditions (RCs) was defined by combining information on personal health status, age, and conditions of cohabiting. In particular “risk condition” (RC) was labeled as “yes” for people who are aged 65 or older, have at least one chronic condition from those listed, or live with chronic patients or with children under the age of 9. Moreover, the intention to get the influenza vaccine was dichotomized as “very or fairly high” vs. “slightly or not at all”. The association between the intention to get the influenza vaccine and the other collected variables (sex, age, qualification, and risk conditions) was tested using the Chi2 test. Moreover, Pearson’s correlation analysis was conducted between the VCI and the HLVa-IT score, with the latter considered either as a total scale or in its components (HLVa-IT-F and HLVa-IT-ICC subscales). 

**Figure 1:**
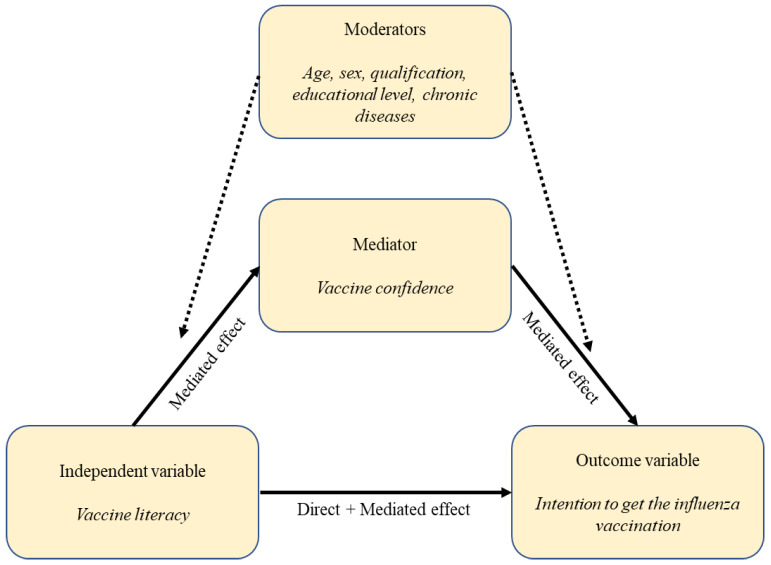
Hypothesized causal pathway of vaccine confidence as a mediator of the association between vaccine literacy and the intention to get vaccinated against influenza.

A model-based causal moderated mediation analysis was performed. In mediation analysis, a pathway is specified a priori, in which an independent variable of interest influences an outcome through an intermediate variable, which is referred to as a mediator. Specifically, the causal pathway was aimed at assessing whether VC mediates the association between the intention to get vaccinated and VL (Figure 1). Age, profession, and risk condition were considered as moderators of the relationship between VL and the intention to get vaccinated against influenza in the 2020–2021 season. When performing the analysis for the mediator model, staff members who were not eligible for the estimation of VL due to their responses to the filter questions in the HLVa-IT were excluded from the analysis.

The analysis proceeded In two steps. First, the linear regression models were separately fitted to investigate the association between the exposure, i.e., VC, and VL (“mediator model”) and between the exposure and the outcome (intention to get vaccinated against influenza in the 2020–2021 season, dichotomized as previously described); the latter was the “outcome model”, which also includes the mediator as a covariate. Separate analyses were performed for the risk condition categories (yes/no) and considering either the VL measure as the HLVa-IT score or in its components (HLVa-IT-F and HLVa-IT-ICC subscale scores). 

Second, the outputs of the mediator and outcome models were used to feed a mediation model fitted by using the “ldecomp” command in Stata software (version 15). The mediate function computes the total effect of the exposure on the outcome and decomposes it into an indirect effect (average causal mediation effect—ACME) and a direct effect (average direct effect—ADE). The analysis was repeated according to the moderator levels. As to the interpretation, the indirect effect reflects the magnitude of the effect that is transmitted through the mediator, whereas the direct effect accounts for all the other possible causal chains. In addition, the “mediate” command yields the “proportion mediated”, which should be interpreted as an estimate of the percentage of the total effect that is exerted through the mediator (Figure 1). 

A mediation model was performed, including only the HLVa-IT subscales significantly associated with the VCI. The VCI was included in the model as a continuous variable, while the subscales of VL were categorized into three levels according to tertials and classified as low, medium, and high.

For each analysis, an α level below 0.05 was considered significant.

## 3. Results

As a whole, 1794 people filled out the questionnaire and were eligible for calculating the scores at the HLVa-IT. The collected data are described in Table 1. 

The respondents were mainly females (86.3%) and assistants/aides (58.1%). The median age of the total sample was 46 years (IQR: 36–53). The respondents living with children, elderly people, or people with chronic conditions made up 42.3%, while those suffering from at least one disease considered at-risk for influenza made up 15.9%. Overall, 759 (42.3%) of the respondents were living with at-risk conditions (either considering age >64 years, their own diseases, or living with people at risk). Regarding influenza vaccination, 55.5% reported their intention of getting vaccinated as “very high” or “fairly high” in the 2020–2021 season; it was significantly higher (Chi2 = 14.4625, *p*-value < 0.001) among respondents with risk conditions (59.9%) with respect to those without risk condition (50.7%). 

The intention to take up influenza vaccination was significantly associated with age (the higher the age of the respondents, the higher the percentage of those who reported their intention of getting vaccinated as “very high” or “fairly high”). Additionally, there was a significantly higher percentage of respondents declaring willingness to get the vaccine (“very high” or “fairly high”) among clinical staff, those living with elderly individuals, or people with chronic diseases, and those who had diabetes, autoimmune diseases, a respiratory infection in the past year, or at least one of the listed diseases. When considering the new variable “risk condition”, the intention to get vaccinated was significantly higher among those classified as “yes” (Table 1).

Concerning VL, calculated based on 1023 subjects (771 were excluded from the mediator model because of their response to the screening question in the HLVa-IT) the median HLVa-IT was 3.1 (IQR: 2.7–3.5; 33rd centile: 2.6; 66th centile: 2.9), the median for the HLVa-IT-F subscale score was 1.8 (IQR: 1.2–2.2; 33rd centile: 1.4; 66th centile: 2.0), and the median for the HLVa-IT-ICC subscale score was 3.2 (IQR: 2.8–3.5; 33rd centile: 2.9; 66th centile: 3.5).

Table 2 describes agreement or disagreement with respect to certain statements about knowledge, attitudes, and beliefs about influenza, vaccinations in general, and influenza vaccination of the NH staff, which were used to calculate the VCI. About 58% partially agreed that influenza is generally a risky disease; 69.6% were in total agreement that COVID-19 is a risky disease for NH health workers. 

### 3.1. The Mediator Model

#### 3.1.1. Association between Vaccine Literacy and Vaccine Confidence (Mediator Model)

Among all respondents, 771 were excluded from the mediator model because of their response to the screening question in the HLVa-IT. A significant association was observed between the intention to get vaccinated and both the VCI and the HLVa-IT-ICC subscale scores in the total sample of participants and for respondents with an RC. Among respondents without an RC, only a high level of HLVa-IT-ICC (with respect to medium or low levels) is associated with the intention to get vaccinated. The HLVa-IT-F scores were not correlated with the VCI (Rho = −0.09; *p* > 0.05), while the HLVa-IT-ICC scores were positively associated with an increase in the VCI (Rho = +0.254). For this reason, the moderated mediation analysis was performed including only the HLVa-IT-ICC. The results of the mediator models are reported in Table 3. VCI is negatively associated with the HLVa-IT-ICC subscale score, either considering respondents with or without an RC. In particular, for respondents without an RC, only the higher level of the HLVa-IT-ICC subscale score (i.e., higher than 3.475) has an impact on the VCI value.

#### 3.1.2. Association between Intention to Get Vaccinated, Vaccine Literacy, and Vaccine Confidence (Outcome Models and Mediation Analysis) 

All of the mediation models showed a significant mediation role of VCI values in the relationship between HLVa-IT-ICC subscale scores and the intention to get vaccinated, either in the total sample or in all of the subsamples considered (see Table 3).

The effects of high and medium versus low HLVa-IT-ICC subscale scores on the intention to get vaccinated are mediated by the VCI values. In particular, respondents with low or medium HLVa-IT-ICC subscale scores would increase their intention of getting vaccinated (OR = 2.22 and 1.59, respectively) if they had the same VCI values as respondents with high HLVa-IT-ICC subscale scores. No direct effect of the HLVa-IT-ICC subscale score on the intention to get vaccinated was observed after considering the mediator effect of the VCI. Regarding the mediation analysis by an RC subgroup presented in Table 4, it was observed that:

- For staff members with at least one risk condition, the total and mediated effects were significant only when considering high or medium HLVa-IT-ICC subscale scores compared to low scores;
- For staff members without risk conditions, the total and mediated effects were significant only when comparing high subscale scores to medium or low scores.

**Table 4:** Mediation analysis by risk conditions subgroup.

Moderated Mediation Analysis *	Risk Conditions (N = 530)				No Risk Conditions (N = 493)			
*High or Medium vs. Low HLVa-IT-ICC*	OR	95% CI		*p*	OR	95% CI		*p*
Total Effect	1.79	1.18	2.71	0.006	1.48	0.98	2.25	0.064
Mediated effect	1.94	1.50	2.52	<0.001	1.82	1.37	2.41	<0.001
Direct effect	0.92	0.68	1.24	0.583	0.82	0.66	1.01	0.058
Moderated mediation analysis *	**Risk conditions (N = 530)**				**No Risk conditions (N = 493)**			
*High vs. Median or Low HLVa-IT-ICC*	**OR**	**95% CI**		***p***	**OR**	**95% CI**		***p***
Total Effect	1.33	0.98	1.80	0.072	2.19	1.51	3.17	<0.001
Mediated effect	1.53	1.24	1.89	<0.001	1.94	1.52	2.48	<0.001
Direct effect	0.87	0.69	1.08	0.209	1.12	0.86	1.47	0.394

OR: odds ratio; CI: confidence interval. HLVa-IT-ICC: Health Literacy Vaccinale in Italiano—health literacy for vaccination in the Italian language—interactive/communicative/critical subscale (three levels: low for HLVa-IT-ICC score < 2.9; medium for 2.9 ≤ HLVa-IT-ICC score < 3.5; high for HLVa-IT-ICC score ≥ 3.5). * Adjusted for age, risk condition, and qualification.

## 4. Discussion

Although the WHO recommends the influenza vaccine for older individuals, healthcare workers, and those in close contact with at-risk populations, influenza vaccination rates among staff members in NHs tend to be low. Our study reports about 50% of NH staff expressed a high intention to get vaccinated in the 2020–2021 season, and this percentage represents an improvement compared to the previous season [12]. To address this issue, it is important to identify predictors that can inform appropriate interventions. In this study, we aimed to evaluate the role of vaccine literacy (VL) and vaccine confidence (VC) in predicting vaccination intention among NH staff during the period between the first and second COVID-19 pandemics.

Our results showed that vaccination intention was positively associated with both VC and ICC VL but not functional VL. However, when stratified by individual or familial risk conditions, high VL was only associated with vaccination intention among respondents without risk conditions. The mediation analysis showed that VC completely mediated the effect of ICC VL on vaccination intention. Among NH staff with risk conditions, medium ICC VL had an effect on vaccination intention, while high ICC VL was required for those without risk conditions.

**Figure 2:**
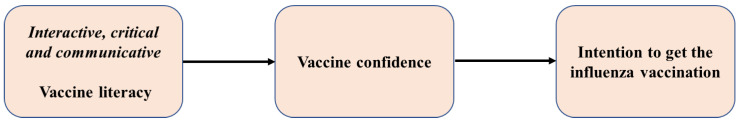
Relationship between VL, VC, and the intention to get vaccinated against influenza according to the results.

The results of our study provide important insights into the factors that affect the intention to get vaccinated against influenza among staff members of NHs: the finding that VC and VL are positively associated with vaccine intention highlights the importance of addressing these factors in efforts to increase vaccination rates among these people. Moreover, the mediation analysis shows that VC is a key factor that mediates the effect of VL on vaccine intention. These results suggest that—despite being specifical medical training on vaccines important for healthcare professionals working in every setting [30]—interventions aimed at improving VL alone may not be sufficient to increase vaccine uptake unless VC is also addressed. Considering that it is known that HCWs with more knowledge and competencies about vaccines are also more likely to recommend vaccination [31], this is a rather important and new result that highlights the need to not only improve their knowledge but also adopt varied strategies to target VC as a whole in this group. On the other hand, the results suggest that interventions aimed at increasing interactive, critical, and communicative VL could have an effect on the increase of VC and then on enhancing the intention to get vaccinated against influenza, as summarized in Figure 2. From this perspective, VL has to be considered as a distal determinant of the intention to get vaccinated against influenza, while VC is a proximal determinant.

Moreover, our results suggest that the presence of individual or familial risk conditions has an effect on the relationship between VL, VC, and the intention to get vaccinated against influenza; compared to NH staff members with risk conditions, a higher level of ICC VL is needed to increase vaccination intention among those without risk conditions. The different impact could be due to the higher information level as well as the higher intention to get vaccinated described for people with individual or familial risk conditions [12,13,26,32].

Successful strategies to overcome vaccine hesitancy that were reported by a recent systematic review demonstrated significant improvement in the utilization of immunization services, and include community-based interventions, monetary incentives, and interventions to make vaccines more accessible [33]. Additionally, reminding healthcare professionals and nursing home staff that influenza can pose a risk to themselves and their families due to illnesses or social conditions is known to have a positive impact on increasing vaccine acceptance in this group [34]. Even when only knowledge is targeted, several studies suggest that programs may require an approach that is tailored to the profession and specific, as knowledge levels and attitudes towards influenza vaccination varied greatly among the different occupational categories [35,36,37]. On the other hand, to the best of our knowledge, the impact of all of these interventions in increasing the interactive, communicative, and critical VL of NH staff members has not been assessed yet. Overall, these results emphasize the importance of addressing both VL and VC in endeavors to enhance influenza vaccine uptake among NH staff. Particularly, increasing awareness and improving preventive behaviors towards influenza viruses gains paramount significance considering the challenges posed by the high mutability of the virus [38,39]. By advancing vaccine uptake, we can effectively safeguard the health of both staff members and residents. This, in turn, contributes to the overall well-being of the community and directly and indirectly aids in elevating vaccination coverage rates, not only within the NH setting but also in the general population [40].

Our study has a number of limitations. First of all, participation was voluntary, either for the directors or for the staff members of NHs, with limitations in the generalizability of the results. Second, the data were self-reported, so we cannot exclude self-reporting bias, which may influence the accuracy of the data, and social desirability bias. On the other hand, the online questionnaire was anonymous, with no possibility to trace who filled it in; for these reasons, we suppose that social desirability bias should be limited. Moreover, calculating the total number of employees of the 98 participating NHs was not possible. This complexity arises from the diverse employment models, wherein some employees are directly hired by the NH, while others are employed by third parties (e.g., cooperative societies that contract nurses/aides and assign them to an NH through agreements). Furthermore, while our study context offers insights into staff vaccination, we recognize the limitations in generalizing the findings to other regions or countries with different contexts and NH organizations. Finally, we acknowledge the absence of information regarding reasons for non-response and characteristics of non-responders, potentially impacting the results’ interpretation.

Nonetheless, to the best of our knowledge, this is the first study conducted in a large sample of NH staff members investigating VL, VC, and the intention to get vaccinated against influenza by using validated measurement scales. 

## 5. Conclusions

Our study examined the predictive role of VL and VC in determining the intention to take up influenza vaccination among nursing home staff members. The findings demonstrated that both VC and VL positively influenced vaccine intention. Specifically, VC was identified as a mediator between the interactive/communicative and critical aspects of VL and vaccine intention. This suggests that interventions focusing solely on improving VL may not be sufficient to enhance vaccine uptake unless VC is also addressed. Conversely, interventions targeting the interactive, critical, and communicative aspects of VL have the potential to impact VC and subsequent vaccination intention. Understanding which interventions are most effective in improving these VL components is crucial for increasing influenza vaccine uptake among nursing home staff, thereby safeguarding their health as well as the health of the elderly residents.

## Figures and Tables

**Table 1:** Sociodemographic and health characteristics of the NH staff (N = 1794) and reported influenza vaccination uptake in the 2018–2019 and 2019–2020 seasons.

Variables		Sample		Vaccination Intention		
		N	%	N	% of Sample	*p* ^c^
Total		1794	100	995	55.5%	
Sex	Males	230	12.8%	138	60.0%	0.163
	Females	1548	86.3%	853	55.1%	
	NA ^b^	16	0.9%	4	25.0%	
Age (years)	<40	551	30.7%	282	51.2%	<0.001
	40–49	519	28.9%	261	50.3%	
	50–59	506	28.2%	310	61.3%	
	60+	218	12.2%	142	65.1%	
Living with	Children of younger than 9 years of age	357	19.9%	200	56.0%	0.525
	Elderly people (>65 years)	358	20.0%	224	62.6%	0.001
	People with chronic diseases	285	15.9%	196	68.8%	<0.001
	At least one of the previously listed conditions	759	42.3%	452	59.6%	0.003
Qualification	Clinical staff	441	24.6%	265	60.1%	0.021
	Assistants/aides	1042	58.1%	551	52.9%	
	Other non-clinical staff	285	15.9%	167	58.6%	
	NA ^b^	26	1.4%	12	46.2%	
Suffering from	Cardiovascular chronic diseases	35	2.0%	24	68.6%	0.115
	Respiratory chronic diseases	137	7.6%	91	66.4%	0.007
	Renal chronic diseases	9	0.5%	8	88.9%	0.043
	Diabetes	31	1.7%	25	80.6%	0.004
	Autoimmune diseases	108	6.0%	73	67.6%	0.009
	A respiratory infection in the past year	48	2.7%	36	75.0%	0.006
	At least one of the previously listed diseases	285	15.9%	189	66.3%	<0.001
Risk condition ^a^	Yes	890	49.6%	533	59.9%	<0.001
	No	818	45.6%	415	50.7%	
	NA ^b^	86	4.8%	47	54.7%	

^a^ Suffering from one disease (or more), age 65+, living with children younger than 9 years of age, elderly people, or people with chronic diseases); ^b^ NA: not available (missing data); ^c^ Chi2 test.

**Table 2:** Agreement or disagreement with respect to certain statements about knowledge, attitudes, and beliefs about influenza, COVID-19, vaccinations, and the influenza vaccination of the NH staff (items included in the VC index).

Items	N (%) Total: 1794				
	Totally Disagree	Partially Disagree	Partially Agree	Totally Agree	Not Available
Influenza is a risky disease	82 (4.6)	179 (10.0)	1045 (58.2)	478 (26.6)	10 (0.6)
It is better to get influenza rather than vaccinate myself	558 (31.1)	339 (18.9)	713 (39.7)	163 (9.1)	21 (1.2)
The influenza vaccine has serious side effects	554 (30.9)	412 (23.0)	686 (38.2)	121 (6.7)	21 (1.2)
The influenza vaccine can cause influenza disease	332 (18.5)	293 (16.3)	805 (44.9)	342 (19.1)	22 (1.2)
The influenza vaccine is effective at preventing influenza disease	73 (4.1)	150 (8.4)	889 (49.5)	670 (3.3)	12 (0.7)
I am against vaccinations	779 (43.4)	235 (13.1)	570 (31.8)	186 (10.4)	24 (1.3)
Healthcare workers should be vaccinated against influenza	161 (9.0)	240 (13.4)	681 (37.9)	700 (39.0)	12 (0.7)
By vaccinating myself, I protect the people I come in contact with from influenza	145 (8.1)	189 (10.5)	627 (35)	819 (45.6)	14 (0.8)

In total, 89% believed that getting sick with influenza could worsen the symptoms of COVID-19 while 76% believed that influenza vaccination could help control and monitor COVID-19 cases. Regarding the VCI, the median score was 1.7 (IQR: 1–2.2).

**Table 3:** Mediator models: Association (linear regression analysis) between the vaccine confidence index score (dependent variable: continuous) and the HLVa-ICC score (three levels: low for HLVa-IT-ICC score < 2.9; medium for 2.9 ≤ HLVa-IT-ICC score < 3.5; high for HLVa-IT-ICC score ≥ 3.5).

Mediator Model Dependent Variable: Vaccine Confidence *	Total (1023)				Risk Conditions (530)				No Risk Conditions (493)			
HLVa-IT-ICC	Coeff.	95% CI		*p*	Coeff.	95% CI		*p*	Coeff.	95% CI		*p*
Low	1				1				1			
Medium	0.20	0.07	0.33	0.002	0.30	0.12	0.48	0.001	0.11	−0.07	0.29	0.227
High	0.54	0.42	0.67	<0.001	0.59	0.41	0.78	<0.001	0.50	0.32	0.68	<0.001
**Outcome model** Dependent Variable: Vaccine Intention *	**OR**	**95% CI**		***p***	**OR**	**95% CI**		***p***	**OR**	**95% CI**		***p***
Vaccine Confidence	13.32	10.12	17.53	<0.001	19.85	11.67	33.77	<0.001	11.40	7.11	18.27	<0.001
HLVa-IT-ICC												
Low	1.00				1.00				1.00			
Medium	1.49	1.10	2.01	0.010	2.33	1.51	3.60	<0.001	0.94	0.61	1.44	0.782
High	1.92	1.41	2.63	<0.001	2.15	1.39	3.33	0.001	1.75	1.13	2.73	0.013
Moderated mediation analysis *	**Total (1023)**				**Risk conditions (530)**				**No Risk conditions (493)**			
*Medium vs. Low HLVa-IT-ICC*	**OR**	**95% CI**		***p***	**OR**	**95% CI**		***p***	**OR**	**95% CI**		***p***
Total Effect	1.49	1.13	1.96	0.005	2.16	1.45	3.21	<0.001	1.00	0.67	1.47	0.982
Mediated effect	1.37	1.14	1.65	0.001	1.62	1.23	2.13	0.001	1.15	0.94	1.42	0.167
Direct effect	1.08	0.87	1.35	0.480	1.33	0.96	1.86	0.089	0.86	0.61	1.21	0.393
*High vs. Low HLVa-IT-ICC*	**OR**	**95% CI**		***p***	**OR**	**95% CI**		***p***	**OR**	**95% CI**		***p***
Total Effect	1.98	1.47	2.68	<0.001	2.21	1.34	3.65	0.002	1.78	1.21	2.62	0.003
Mediated effect	2.22	1.80	2.73	<0.001	2.40	1.67	3.46	<0.001	2.05	1.59	2.65	<0.001
Direct effect	0.89	0.72	1.10	0.294	0.92	0.68	1.25	0.593	0.87	0.62	1.22	0.411
*High vs. Medium HLVa-IT-ICC*	**OR**	**95% CI**		***p***	**OR**	**95% CI**		***p***	**OR**	**95% CI**		***p***
Total Effect	1.34	0.99	1.80	0.057	1.03	0.62	1.71	0.922	1.79	1.28	2.50	0.001
Mediated effect	1.59	1.28	1.98	<0.001	1.40	0.99	1.96	0.055	1.83	1.42	2.36	<0.001
Direct effect	0.84	0.67	1.05	0.120	0.74	0.54	1.00	0.052	0.98	0.73	1.32	0.888

OR: odds ratio; CI: confidence interval; VCI: vaccine confidence index. HLVa-IT-ICC: Health Literacy Vaccinale in Italiano—health literacy for vaccination in the Italian language—interactive/communicative/critical subscale. * Adjusted for age, risk condition, and squalification.

## Data Availability

Data supporting the reported results are available from the corresponding author upon request.

## Supplementary Materials

The following supporting information can be downloaded at: https://www.mdpi.com/article/10.3390/vaccines11081375/s1. Supplementary File S1: HLVa-IT.
