# Supplementary material for: Does Vaccine Confidence Mediate the Relationship between Vaccine Literacy and Influenza Vaccination? Exploring Determinants of Vaccination among Staff Members of Nursing Homes in Tuscany, Italy, during the COVID-19 Pandemic
Source: Vaccines (Basel). 2023 Aug 17;11(8):1375. doi: 10.3390/vaccines11081375 (PMC10458978; doi:10.3390/vaccines11081375)
Supplement: Supplementary file 1 [file vaccines-11-01375-s001.zip › vaccines-2555101-supplementary.pdf]

Supplementary File S1: HLVa-IT

HLVa-IT

"Have you ever read vaccine materials, such as leaflets or posters in doctor's or public health units offices, recommending vaccinations?"

□ NO □ YES

If yes, fill in the box below, marking with an X the boxes corresponding to your choice (choose only one answer for each question)

READING THE MATERIAL:	Never	Rarely	Sometimes	Often
1. Did you find that the material as a whole (texts and/or images) was difficult to read?	□	□	□	□
2. Did he find words you didn't know?	□	□	□	□
3. Did you find that the texts were difficult to understand?	□	□	□	□
4. Did you need much time to understand them?	□	□	□	□
5. Did you or would you need someone to help you understand them?	□	□	□	□

"Have you ever thought or been advised to vaccinate yourself against one or more diseases?"

□ NO □ YES

If yes, fill in the box below, marking with an X the boxes corresponding to your choice (choose only one answer for each question)

WHEN SEARCHING INFORMATION:	Never	Rarely	Sometimes	Often
6. Have you consulted more than one source of information?	□	□	□	□
7. Did you find the information you were looking for?	□	□	□	□
8. Did you understand the information found?	□	□	□	□
9. Have you had the opportunity to use the information?	□	□	□	□
10. Did you discuss what you understood about vaccinations with your doctor or other people?	□	□	□	□
11. Did you consider whether the information collected was about your condition?	□	□	□	□
12. Have you considered the credibility of the sources?	□	□	□	□
13. Did you check whether the information was correct?	□	□	□	□
14. Did you find any useful information to make a decision on whether or not to get vaccinated?	□	□	□	□
